# Obstetrics, Gynecology and Pelvic Surgery

**Published:** 1895-10

**Authors:** William Warren Potter

**Affiliations:** Examiner in obstetrics, New York state medical examining and licensing board; consulting gynecologist to the Buffalo Woman’s Hospital


### OBSTETRICS, GYNECOLOGY AND PELVIC SURGERY.

CONDUCTED BY WILLIAM WARREN POTTER, M. D.,

Examiner in obstetrics, New York state medical examining and licensing board ; consulting gynecologist to the Buffalo Woman's Hospital.

### THE OBSTETRIC BINDER.

WINDMUELLER (*Centralbl. f. Gynäk.*, quoted in *Medical Review*,) recently opened a long and instructive discussion on the management of normal labor and childbed. He had conducted over a thousand labors in private practice alone in the course of thirty years. He wraps flannel around the body, but objects to the abdominal binder, which he finds interferes with the functions of the intestines, checks involution of the uterus, and is in the way during ablution. For the first three days he limits diet to milk, bread and butter, rice, spinach, potatoes and fish. He forbids wine and coffee and believes, with Zweifel, that errors in diet are among the chief causes of prominent abdomen. The patient is kept on her back for three days, but Windmueller holds that ten days' rest in bed is sufficient. He has had no cases of prominent abdomen in any of his patients even in multiparæ, with the exception of those who had grown fat. In the discussion, several experienced obstetricians differed as to whether prominent abdomen was due to deposit of fat in the parietes after labor, or to distention of intestines which could be controlled by the binder. Many surgeons applied no bandages after abdominal section; but the physiological conditions were not the same as in the puerperium. Schrader and others believed that the binder did more good than harm; he laid great stress on diet in childbed as influencing the future form of the abdomen. All food liable to cause flatulence must be avoided; spinach and potatoes were particularly bad in this respect.

When the pelvis was narrow, or its angle too acute, the chances of the abdomen becoming pendulous after labor were great. To counteract the evil, a good abdominal binder should be worn during the last months of pregnancy. In Japan it was customary for women to wear a binder during the entire second half of gestation. The binder in pregnancy supported the anterior walls, and greatly diminished the chances of parting of the recti.

---

### A FEW POINTS IN OBSTETRICS.

No BRANCH of a physician's practice requires more self-poise, where so many complications arise, commanding our sympathy and demanding our skill, than that of obstetrics, and when disease and death follow a normal case of labor, the cause can be traced to none other than to ignorance or mismanagement.

With these preliminary remarks, Ewing (*Med. Review*, quoted in the *Lancet-Clinic*,) advances a few aphorisms relating to obstetrics.

1. Examine the urine a week or so before the expected confinement. Albumen need not cause alarm, unless present in large quantity, in which case the woman should be restricted to milk diet, given 1-10 grain of sulphate sparteine four times a day, and bowels kept open with cream of tartar, the object being, of course, to relieve congestion of the renal veins.

2. Make no digital examination without first cleansing the hands and nails, together with the external genitals, with a solution of bichloride of mercury (1 to 2,000) and ethereal soap.

3. Empty the rectum thoroughly with an injection of warm water.

4. Make as few examinations as possible during progress of labor, and each time dip the hand first in the antiseptic solution.

5. If the presenting part emerges slowly from the womb, do not allow your impatience to so get the better of your judgment as to induce you to "assist nature" by pulling upon the os. Probably all the deep pathological tears, calling for surgical interference, found on the right and upper anterior sides of the cervix, are caused by the finger of the accoucheur.

---

### CESAREAN SECTION.

We read in the *Dublin Journal of Medical Science* (*Clinique*, St. Louis,) that the first successful case of Cesarean section on the living patient performed in the three kingdoms, was performed in January, 1738, by a handy-woman, Mary Dunally, with a razor, on Alice Neal, at Charlemont, between Armagh and Dungannon. Twenty-seven days after the operation, the patient was able to attend Armagh market, walking to and from the city with her marketing.

---

### PREGNANCY WITH UNRUPTURED HYMEN.

Guerard (*British Medical Journal*, quoted in *Maryland Medical Journal*,) relates three new cases of pregnancy in which the hymen was persistent. In the first and second there was a protracted second stage due to the resistance of the hymen, which was perfect and very elastic. After a crucial incision the fetus was at once delivered, but in one case the child was lost. In the third case the patient appeared to be in the seventh month of her first pregnancy, and suffered from severe pain in the genital tract. Although she had twice been operated on for atresia of the hymen, the vagina was still closed by a firm, impermeable and tender membrane. This was excised, the pains disappeared and the pregnancy continued and ended naturally. Guerard notes a case of bifenestrated hymen where the openings barely admitted a hair ; yet the patient reached the third month of pregnancy, and abortion was induced in a manner which could not be ascertained. In considering these cases, he notes how the alkaline uterine mucus, poured out during orgasm, protects the spermatozoa from destruction by vaginal mucus.

### RECTAL EXAMINATION OF PREGNANT WOMEN.

W. H. Beckman (*Shurnal akuocherstwai Shenskich bolesnej*, quoted in the *Med. and Surg. Rep.*,) has tried this method with great success in 100 parturient women, the details of pelvis and cervix being easily made out. The length of the pregnancy and state of the bladder could not be determined in 7 per cent. of the cases, and the fontanelles and sutures could not be felt in 28 per cent., but this was less important, since the position of the fetus could easily be detected by external examination and especially by auscultation. It was always possible to distinguish the occipital from the frontal portion of the head. The advantage of rectal examination is that infection through the genitals is avoided, the only objection being that sometimes examination through the vagina may become necessary, and that infection may occur through the examining finger, though the latter is easily disinfected, since the rectum does not contain specific microbes. The author considers rectal examination of great value to midwives, enabling them to determine if the presence of an obstetrician will be necessary. Zweifel's experience in the Leipzig obstetrical clinic showed that students instructed in this method of examination could determine all the necessary details without recourse to vaginal examination. Ries believes that midwives should be forbidden to make examinations through the vagina, as their duty is only to assist at normal births. Kroenig is inclined to permit vaginal examinations only (1) when it is difficult to determine through the rectum what part of the fetus is presenting ; (2) when the midwife is not able to bring about relaxation of the cervix and (3) when the pains last more than two hours.

---

### UNCONTROLLABLE VOMITING OF PREGNANCY.

Purch (*Mod. Med.*, quoted in *Med. and Surg. Rep.*,) recently introduced a discussion on this question before the Société Obstetricale de France. His patient, a hystero-neurasthenic subject, suffered very early, so he emptied the uterus, by the curette, at the sixth week, without loss of blood. The vomiting at once ceased. Circumstances contraindicated palliatives in this case. Gaulard believed that palliatives should be tried until there was a rise of temperature, which was a dangerous symptom. Charpentier stated that he is now in favor of terminating the pregnancy as early as possible. During the first three months it is easy to get all the ovum away. He now introduces into the uterus a short stick of solid nitrate of silver, which is left to melt there. But in one case where a practitioner, fearing to use so strong a caustic, introduced a pledget of cotton soaked in 20 per cent. solution of nitrate of silver, the result was prompt and satisfactory. Disproportion between pulse and temperature is a grave symptom. Marduel related a serious case. The patient had an insane blood relation. Bad vomiting set in during her pregnancy. Opium was given, and she then could keep a little food down, but symptoms of abortion appeared, the drug having probably killed the fetus. Partial blindness set in. A laminaria tent was introduced; next day Fochier emptied the uterus under ether. This proceeding proved very difficult, the uterine walls and even the abdominal parieties being bruised. The sickness ceased, but returned on the next day. A trace of albumin was detected in the urine. For a week the patient grew worse, becoming quite blind from retinal hemorrhage and edema around the disc due to cerebral mischief. A blister was applied to the nape. The sight was partly restored, and a few days later the vomiting ceased. Marduel's services were dispensed with too early; he was recalled and found the patient delirious. She died on the same day, nearly a month after the induced abortion. Fochier, in reference to the difficulty of emptying the uterus when he operated in Marduel's case, observed that ether is a good anesthetic for such work. The uterus must be well depressed when the finger is used to empty the uterus, as the placenta is very adherent in these cases.

---

### THE FILLET IN BREECH LABORS.

Bar (*Arch. de Tocol. et de Gynec.*, quoted in *Med. and Surg. Rep.*,) exhibited, at the February meeting of the Paris Obstetrical and Gynecological Society, an infant, aged 2, with a deep scar on the right groin. It limped slightly and the right thigh was four-fifths of an inch short. The mother at its birth was a primipara, aged 39; the child was then very bulky, and the fillet was used, as all other means to deliver the child, especially the forceps, had failed. Bar attributed the shortening to atrophy of the head of the femur following separation of the epiphysis due to the fillet. He exhibited a similar case where he had used the fillet. A deep incised wound lay in the left groin. A crack was heard during the extraction. The wound suppurated and the child died of pneumonia. Charpentier admitted that he had damaged both soft parts and bones even when employing the fillet with the greatest care. Gueniot always aided the traction of the instrument by passing the hollow of the hand into the concavity of the sacrum and exercising further traction. This provoked uterine efforts. Budin added uterine expression as an aid to the fillet. Porak believed in the application of two fillets, one on each thigh. Maygrier held that the fillet should be used in dorso-anterior, and the forceps in dorso-posterior, positions. By that principle, fractures are avoided. Olivier protected the fillet by enveloping it in a rubber tube. In that way he had always avoided accidents.

---

### BIRTH OF A CHILD WITHOUT RUPTURE OF MEMBRANES.

Dr. W. M. Morison reports a case in the *British Medical Journal*, quoted in *Medical and Surgical Reporter:* The patient was suffering from acute phthisis, with a temperature of about 101°. Two days before parturition she complained of severe diarrhea, which was, however, quickly and easily controlled. Some time before, she miscarried at the fifth month, and a day or two prior to it she had a similar attack of diarrhea, which was but the precursor of a second miscarriage. She was in her seventh month of pregnancy. Her surmise was perfectly correct, as in two days after she complained of some pains in the lower part of the abdomen. I was sent for, and on arriving there found a cyst-like mass protruding from the vagina. The labor was absolutely dry, not a trace of blood was to be seen, and the uterus contracted without any trouble. The recovery from the immediate effects was highly satisfactory, considering the advanced stage of phthisis in which the patient was. The membranes enclosing the fetus were quite intact and contained practically no amniotic fluid. The cord was pulseless and no indication of life in the fetus, which was otherwise a well-developed seven months' growth. The placenta was normally attached and the presentation was a breech. Children born with a "caul," as a condition such as this is called, are, when the child survives, a source of great satisfaction to the parents, especially parents of a superstitious bias, and such are very numerous yet, because of a supposed "luck" which smiles upon the path through life of those who make their entrance into this weary world shrouded in the mysterious garb of the mysterious world from which they come. In premature births, is it not a fact that breech presentations are as numerous, or, at any rate, a more frequent occurrence than at full time?

### INDICATIONS FOR INDUCTION OF ABORTION.

Dr. Jeffé, (*Medicinische Neuigkeiten*, No. 45, 1894 ; *Pacific Medical Journal*,) from a study of the literature of the last ten years, fixes the indications for inducing abortion as follows :

*Absolute Indications.*—(1) Uncontrollable vomiting of pregnancy ; (2) incarceration of the gravid uterus ; (3) obstruction of the pelvic outlet by tumors or exudates ; (4) progressive and pernicious anemia ; (5) grave chorea.

*Relative Indications.*—(1) Great contraction of the pelvis with the conjugata vera below 5 cm. ; (2) pulmonary emphysema with signs of degeneration of the heart ; (3) nephritis ; (4) chronic heart disease ; (5) other general diseases of the mother which would jeopardise her life at the time of delivery.

He holds that a conjugata vera of 6 cms. and advanced pulmonary tuberculosis should not be regarded as indications for abortion. He does not think it just to sacrifice a future life for one that is "certainly lost."

---

### THE ABSORPTIVE POWER OF THE VAGINA.

From a series of experiments conducted upon pregnant and puerperal women, upon those suffering from fever and diseases of the genitalia, and upon women convalescing from hysterectomy, Coen and Levi (*Collegiene Ital. di Litture Sulla Med.*, serie vii., No. 2 ; *Phil. Polycl.*,) conclude that the vagina undoubtedly has absorptive powers, and that these powers are increased in pregnancy, in the puerperium and in fevers. Among the drugs experimented with, salicylic acid, salol and phenazone were found to be rapidly absorbed ; potassium iodid readily, and iodoform in small quantities. One hour after the insertion of a tampon saturated with a 20 per cent. solution of potassium iodid, the urine contained iodin. The absorption was very rapid in fever patients, but unaltered after hysterectomy. To secure rapid absorption of iodoform, the vagina should be insufflated with fresh iodoform, which is then allowed to remain several days. Phenazone appears in the urine in one and a half hours and remains for forty-eight hours. Its antipyretic action, however, is less than when administered by the mouth. Salicylic acid appears in the urine in one hour and persists for twenty-four hours. Salol also appears early and traces of it appear in the urine for some time.

### INOPERABLE UTERINE CARCINOMA.

Lucas, in the *Rev. Int. de Méd. et de Chir. Prat.*, (*Phil. Polycl.*, December, 1894,) recommends the following powder for arresting and diminishing the fetid discharge and preventing vulvar and perineal excoriations : Two drams each of benzoin, iodoform and magnesium carbonate. This should be freely applied to the carcinomatous tissue.

---

### METHYL-BLUE IN PRURITUS VULVÆ.

Madden (*La Méd. Mod.*, March 9, 1895 ; *Phil. Polycl.*,) recommends, in pruritus vulvæ, a 10 per cent. lotion of methyl-blue after thorough bathing of the parts in warm water and corrosive sublimate solution (1 to 1000). In addition, he administers internally the same drug in doses of 1½ grains, in capsules, twice or thrice daily. He claims marked improvement under this mode of treatment, but admits that the skin to which the preparation is applied is permanently discolored.

---

### PHENAZONE IN PRURITUS.

Blaschko, in 1891, recommended the employment of phenazone internally for the correction of pruritus, either general or local. F. Arnstein (*Gazeta Lekaeska*, No. 48, 94 ; *Phil. Policl.*,) has adopted this suggestion in his severe cases, with excellent results. The first patient was a young woman, twenty-eight years of age, with pruritus nervosis of three months' standing ; the second was a woman of sixty-six years, with chronic pruritis senilis. In both, the itching quickly subsided and in two weeks' time had entirely disappeared. All other known remedies had failed in each instance.

---

### SUDDEN DEATH DURING COITUS.

At a meeting of the Biological Society, M. Luys (*La Trib. Méd.*) exhibited sections of the brain of a young officer who died during a coitus which took place after he had dined. The sections revealed the presence of a series of small, sanguineous extravasations, without rupture of the vascular walls, in the substance of the corpus callosum and island of Reil. The speaker alluded to the danger, even in young persons, of cohabitation after a meal.

### COLD BATHING DURING MENSTRUATION.

DEPASSE (*Gazette de Gynacologie;* quoted in *Dominion Medical Monthly,*) says cold bathing during menstruation is a beneficial measure, provided women accustom themselves to the treatment by bathing every day for at least eight days before the arrival of the period, when they can continue during the menstrual flow without any danger. In the case of a very anemic girl, in whom this treatment was instituted, it gave most satisfactory results. Houzel, before the recent Boulogne Congress, held that cold salt-water baths facilitate the menstrual flow, increase the duration of genital life and likewise increase fecundity in a remarkable manner.

---

### CASE OF CONCEPTION DURING THE PUERPERAL PERIOD.

BRASSEUR (*Centralbl. für Gynecol.; Ont. Med. Jour.,*) quotes the case of a woman, aged 22, who was delivered on July 4, 1892, of her first child. July 8th she practised coitus and was again delivered March 10, 1893, of a child measuring fifty-two centimeters in length and weighing 3,550 grammes. Calculating from the date of coitus, the second pregnancy lasted 243 days, that is, twenty-seven days less than the normal.

The case caused considerable discussion. Ovulation must have existed in the woman on the fourth day after the delivery, and it was necessarily quite independent of menstruation.

Koenig, who originally reported the case, draws from it the following deductions :

1. A gestation period of 243 days after a fecundating coitus may produce a viable child.
2. The spermatozoa can live in the lochial secretions.
3. The functional activity of the ovaries is not completely suspended during pregnancy. The Graafian follicles so open that they may burst a very short time after delivery.
4. Ovulation and menstruation may occur independently of each other.
5. Among vigorous women, during the period immediately following confinement, the uterine mucous membrane may undergo a rapid regeneration which renders possible the implantation of a fecundated ovule immediately after delivery.

---

### OVARIOTOMY, EIGHTY-EIGHT QUARTS OF FLUID—RECOVERY.

REIFSNYDER (*Amer. Jour. of Obstet.,* quoted in *Med. and Surg. Rep.,*) describes two cases of ovariotomy performed on native

Chinese women in the Margaret Williamson Hospital, Shanghai; both patients recovered. In the first case the patient was 23; the tumor weighed eighty pounds. The second patient was 25 years of age; she married at 19, and soon afterward her abdomen began to enlarge. She had never been tapped. She was four feet, eight inches in height, and the circumference at the umbilicus was five feet, seven and three-fourths inches. She passed about sixteen ounces of urine in twenty-four hours, free from albumin; its specific gravity was 1026. Her appetite was good and the bowels acted once or twice daily. She had been unkindly treated as a sterile woman unfit for domestic work. After numerous precautions, ovariotomy was performed. Chloroform was given; her head and shoulders had to be somewhat elevated; she took but little of the anesthetic. Eighty-eight quarts of fluid were removed; the tumor consisted of one large and one very small cyst; there were free adhesions superiorly. The empty tumor weighed six and one-half pounds. There was no ascitic fluid in the abdominal cavity. The pedicle was long and about two and one-half inches broad. The abdomen was washed out, then the wound closed with twelve silk sutures. There was much shock at first. After the first day, she passed urine voluntarily and her bowels were moved forty-one hours after operation. On the second day, there was flatulent distention, the pulse rising to 102. Rochelle salts were given. Her second night was the worst; she had two hypodermic injections of digitalin (1-100 grain), brandy by the mouth twice, one turpentine enema, and a capsule of turpentine taken by the mouth. Afterwards she did well. Her weight, two months after the operation, was ninety-two pounds.

---

### PATHOLOGY OF VAGINISMUS.

Thos. More Madden stated at the British Medical Association (*Med. Age*), that abnormal sensibility attended with spasmodic contraction of the vulvo-vaginal orifice often comes under observation as a cause of dyspareunia. In such cases the local hyperesthesia is evinced on any attempt at examination, and is most marked about the meatus urinarius or in the vicinity of the vulvo-vaginal glands and fourchette whence the hymen or its remains project upwards.

Without referring here to the different theories which have from time to time prevailed with regard to the general causation of vaginismus, or to the minor cases of this complaint, which are commonly sufficiently relieved by the topical application of methylene blue, cocaine, or other local analgesics, confining myself to the etiology and treatment of those graver forms of vaginismus, occasioning dyspareunia, which require more serious remedial measures, I would presume to express my opinion, founded on somewhat extensive experience, that in the great majority of these instances the complaint is not only very intimately connected with the constitutional neurotic temperament generally evinced by such patients, but also that, hardly less frequently, it is also largely ascribable to a special local lesion—namely, an abnormal condition, or neuritis, affecting the trunk or terminal fibrillæ of the pudic nerve, one branch of which supplies the structures in the vicinity of the clitoris, whilst the other, or superficial perineal nerve, is distributed to the labia as well as to the perineum in which its terminal branches ramify.

---

### ROUND ULCER OF THE VAGINA.

Wlodzimicrz Skowronski (*Univ. Med. Mag.*) describes a case of perforating ulcer of the anterior wall of the vagina in a multipara thirty-seven years of age. It was half a centimeter in diameter, rough, with granulating detritus, gray in color and bleeding on touch. The ulcer was extirpated and the wound closed with silver-wire sutures. The patient, who was anemic, improved in health. Microscopical examination showed an absence of mucous membrane, the submucous layer being partly intact. The vessels in the ulcer had coalesced and their walls were thickened. The prognosis in such cases is regarded as unfavorable by the author, on account of the probable recurrence. Should the ulcer extend to any depth, it is likely to cause fistula or fatal hemorrhage. The treatment of neglected cases is difficult and unsatisfactory. The cause, according to Zahn and Browicz, is local atrophy of the blood-vessels.—*Przeglad Lekarski*, No. 37, 1894.

---

### GONOCOCCI IN THE VAGINAL SECRETIONS.

Buttner, in the space of three months, examined fifty-four prostitutes of Dorpat, to ascertain whether or not they were affected with blennorrhagia. Of these, thirty-two were subjected to a semi-weekly examination through the speculum; the other twentytwo were encountered in the hospital. Of these latter, in only six did microscopic examination show the presence of a blennorrhagia. In eleven of the remaining sixteen, or 68 per cent., bacteriologic examination showed the presence of the gonococcus in the vaginal secretion, and the same result in nine, 28 per cent., of the prostitutes registered by the police. Among the prostitutes under treatment in the hospital, Dr. Buttner made a separate examination of the vaginal secretion, the cervical mucus and the secretion from the urethra. In the eleven women with manifest blennorrhagia he never found the gonococcus in the vaginal secretion. This microbe was found six times in the cervical mucus and the urethral secretion at the same time ; four times in the secretion from the urethra only ; once in the cervical mucus only. The author concludes that the examination of prostitutes, as it is practised at present, does not give sufficient basis for the establishment of a certain diagnosis.—*Journal American Medical Association.*

---

### IMPREGNATION FROM SEMEN DEPOSITED ON THE VULVA.

Dr. R. C. Longfellow, of Cincinnati, says, (*Lancet-Clinic,*) on January 12, 1891, he was consulted by a gentleman who desired a prescription for a lady friend who had " missed " three menstrual periods. The history of the case pointed to pregnancy, but the lady was indignant and said that this was not possible as no intercourse had taken place. Her friend, however, returned and told the doctor that some three months before he had attempted intercourse, but, that owing to the fact that the attempt caused the lady much pain, he had desisted without having succeeded in introducing his penis into the vagina. An emission of semen upon the vulva occurred, however, and the lady left his office without making any toilet, allowing the semen to remain in the vulva over night. This was the only time that coition had been attempted. The lady was persuaded to allow an examination. The vulva presented a virgin condition, a thin hymen present and the vagina so small as to scarcely admit the finger. The evidences of pregnancy were well marked and early in August she was delivered of a male child.

---

### IMPREGNATION OF ONE SEXUAL PERVERT FEMALE BY ANOTHER.

Duhousset (*Moll's Conträre Sexualempfindung,* quoted in *Medical Standard,*) reports the case of two sexual pervert femâles which came under his observation. One of them at length married, but kept up her relations with the other. The unmarried female had an enlarged clitoris by which coitus was performed. The unmarried pervert became pregnant to her own astonishment. The matter was later explained by the admission of the married pervert that, immediately after coitus with her husband, she had indulged with her "friend," who thereby impregnated herself.

